# Bioinformatic Analysis of the Type VI Secretion System and Its Potential Toxins in the *Acinetobacter* Genus

**DOI:** 10.3389/fmicb.2019.02519

**Published:** 2019-11-01

**Authors:** Guillermo D. Repizo, Martín Espariz, Joana L. Seravalle, Suzana P. Salcedo

**Affiliations:** ^1^Departamento de Microbiologia, Facultad de Ciencias Bioquimicas y Farmaceuticas, Instituto de Biologia Molecular y Celular de Rosario (IBR, CONICET), Universidad Nacional de Rosario, Rosario, Argentina; ^2^Laboratory of Molecular Microbiology and Structural Biochemistry, CNRS UMR 5086, University of Lyon, Lyon, France

**Keywords:** comparative genomics, toxins, PAAR proteins, VgrG, type 6 secretion system, *Acinetobacter*

## Abstract

Several *Acinetobacter* strains are important nosocomial pathogens, with *Acinetobacter baumannii* as the species of greatest concern worldwide due to its multi-drug resistance and recent appearance of hyper-virulent strains in the clinical setting. *Acinetobacter* colonization of the environment and the host is associated with a multitude of factors which remain poorly characterized. Among them, the secretion systems (SS) encoded by *Acinetobacter* species confer adaptive advantages depending on the niche occupied. Different SS have been characterized in this group of microorganisms, including T6SS used by several *Acinetobacter* species to outcompete other bacteria and in some *A. baumannii* strains for *Galleria mellonella* colonization. Therefore, to better understand the distribution of the T6SS in this genus we carried out an in-depth comparative genomic analysis of the T6SS in 191 sequenced strains. To this end, we analyzed the gene content, sequence similarity, synteny and operon structure of each T6SS loci. The presence of a single conserved T6SS-main cluster (T6SS-1), with two different genetic organizations, was detected in the genomes of several ecologically diverse species. Furthermore, a second main cluster (T6SS-2) was detected in a subgroup of 3 species of environmental origin. Detailed analysis also showed an impressive genetic versatility in T6SS-associated islands, carrying VgrG, PAAR and putative toxin-encoding genes. This *in silico* study represents the first detailed intra-species comparative analysis of T6SS-associated genes in the *Acinetobacte*r genus, that should contribute to the future experimental characterization of T6SS proteins and effectors.

## Introduction

The *Acinetobacter* genus comprises a heterogeneous group of strictly aerobic Gram-negative bacterial organisms endowed with great metabolic versatility. This genus includes numerous non-pathogenic environmental species as well as some with significant pathogenic potential, notably *Acinetobacter baumannii*, frequently associated with disease in the context of hospital-acquired infections (McConnell et al., 2013). Although infections due to multi-drug resistant (MDR) *Acinetobacter* species are a serious health threat worldwide, the knowledge about the mechanisms that enable them to colonize the host and hospital environments is still scarce (Antunes et al., 2014; Harding et al., 2018).

Bacteria use several secretory mechanisms to export effector proteins into the environment or straight into target cells (Costa et al., 2015; Galán and Waksman, 2018). Among them, the multicomponent type VI secretion system (T6SS), which is structurally related to the cell-puncturing device of the T4 bacteriophage, was described in Gram-negative bacteria, including *Acinetobacter* (Weber et al., 2017; Elhosseiny and Attia, 2018). The T6SS dynamic machinery permits the injection of toxic effector proteins into prey cells in a contact-dependent manner. The concomitant expression of cognate immunity proteins prevents self-intoxication (Benz and Meinhart, 2014; Alcoforado et al., 2015). T6SS effectors with diverse enzymatic activities have been identified, including the membrane-, cell wall-, or nucleic acid-targeting antibacterial effectors and the heterogenous group of eukaryote-targeting effectors (Lien and Lai, 2017).

**FIGURE 1:**
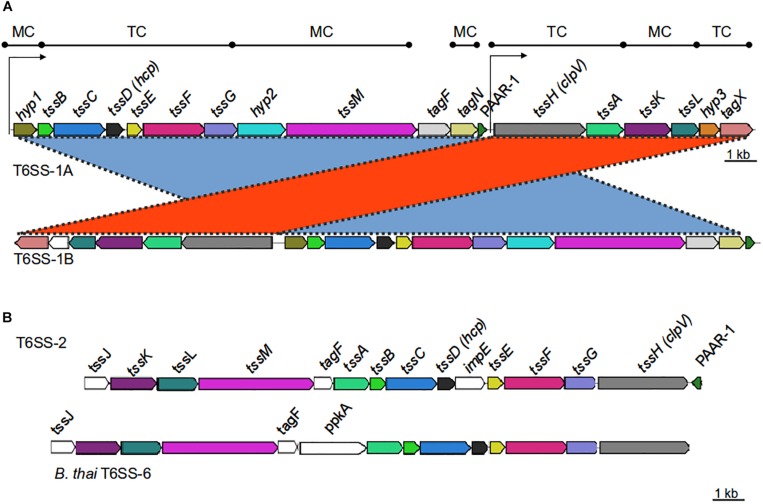
Genetic organization of T6SS-main loci in the *Acinetobacter* genus. **(A)** T6SS-1-main gene cluster (for details refer to Table 1). Gene products are classified according to their participation in the membrane complex (MC) or tail complex (TC) of the T6SS apparatus. Thin arrows represent putative promoter regions. **(B)** T6SS-2-main gene cluster (for details refer to Table 4). *B. thailandiensis* T6SS-6 main cluster is shown for comparison. Genes encoding proteins with amino acidic identity > 25% and query coverage > 50% were represented with the same color.

The T6SS apparatus is assembled from a set of core components proteins (Boyer et al., 2009), which comprise the minimal machinery necessary for its functionality. The genes coding for the core components (*tss*) are frequently organized within one genetic cluster. Most T6SS gene clusters contain additional genes (T6SS-associated genes; *tag*), the function of most remains unknown. Previous studies using a limited number of *Acinetobacter* species predicted the presence of a conserved T6SS gene cluster with two different genetic organizations (Weber et al., 2013). This locus, hereafter referred as T6SS main cluster (T6MC; Figure 1A and Table 1), is described to encompass 18 genes (Carruthers et al., 2013; Weber et al., 2013). Fourteen genes showed a significant homology at the protein level with T6SS components present in other bacteria, and were thus initially named following the nomenclature proposed by Shalom et al. (2007), namel*y t*ss and *tag* genes. The only gene that does not follow this nomenclature is that encoding a PAAR-domain protein, which has been accordingly dubbed just as PAAR gene (Weber et al., 2016). It was recently shown in *A. baylyi* ADP1 that one of the four genes with non-attributable function (ACIAD2699) coded for a peptidoglycan hydrolase (TagX), facilitating the passage of the T6SS apparatus through the cell wall (Weber et al., 2016). Despite the high levels of homology observed between most T6MCs, the genes encoding the VgrG and PAAR-repeat domain proteins (usually more than 1 of each per genome) are scattered throughout the genomes and conservation between *Acinetobacter* strains is low (Eijkelkamp et al., 2014). The regions flanking these genes are known as VgrG and PAAR islands (De Maayer et al., 2011) and represent evolutionary hot spots for genes that encode effector proteins. Genes encoding the cognate immunity proteins are usually present in these regions as well. These regions account for strain specificity regarding T6SS-related components (Unterweger et al., 2014).

**TABLE 1:** T6SS-1-main locus genes in *Acinetobacter* spp.

		***A. baumannii* DSM30011 (T6SS-1A)**		***A. baylyi*. ADP1 (T6SS-1B)**		
**Protein**	**COG**	**Locus (DSM30011_)**	**Length (AA)**	**Locus^a^ (ACIAD)**	**Length (AA)**	**% AA identity/similarity with Ab DSM30011**
Hyp1		11500	229	**2693**	218	52/70
TssB	3516	11495	167	**2691**	169	87/95
TssC	3517	11490	493	**2690**	498	94/96
TssD/Hcp	3157	11485	167	**2689**	167	97/98
TssE	3518	11480	158	**2688**	157	86/94
TssF	3519	11475	603	**2687**	602	73/86
TssG	3520	11470	332	**2686**	332	64/78
Hyp2		11465	470	**2685**	476	43/64
TssM	3523	11460	1274	**2684**	1273	84/92
TagF	3913	11455	319	2683	319	75/86
TagN	2885	11450	255	2682	254	75/86
PAAR	4104	11445	87	2681	88	70/81
TssH/ClpV	0542	11440	892	**2694**	894	80/87
TssA	3515	11435	364	**2695**	367	67/80
TssK	3522	11430	454	**2696**	455	81/91
TssL	3455	11425	268	**2697**	267	81/91
Hyp3		11420	200	2698	193	-
TagX		11415	317	**2699**	287	78/89

**^*a*^Essential components described by Weber et al. (2016) and Ringel et al. (2017) are shown in bold.**

**FIGURE 2:**
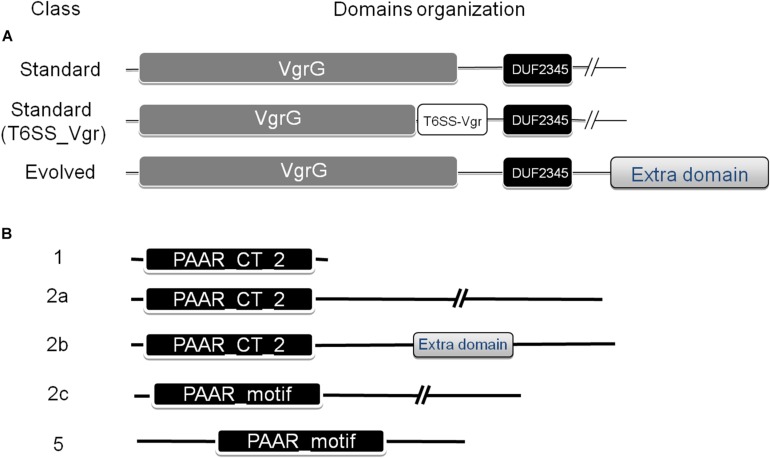
Domain architecture of VgrG **(A)** and PAAR repeat proteins **(B)** found in the *Acinetobacter* genus. The NCBI-CDD (Marchler-Bauer et al., 2015) was used for the domain search. The classification defined by Shneider et al. (2013), which categorized PAAR proteins into seven classes based on domain architecture, was used. Additional subclasses were defined for PAAR-2 proteins (see text for details).

A number of VgrG proteins have been characterized in *A. baumannii* (Weber et al., 2016; Fitzsimons et al., 2018). They all share a common domain structure (Figure 2A), with the typical N-terminal part constituted by domains commonly found in the bacteriophagic components gp44 and gp5, which altogether compose the VgrG domain (COG3501) defining the VgrG superfamily (cl27827). This domain is followed by a DUF2345 domain (pfam10106, superfamily cl27827) close to the C-terminus, responsible for the binding of the effector (Flaugnatti et al., 2016), and a C-terminal stretch of variable aminoacidic composition and length, which could even be totally absent. In *Vibrio cholerae*, the C-terminal portion of a number of these proteins, known as evolved VgrGs, displays an additional domain that functions itself as an effector (Pukatzki et al., 2007; Ma and Mekalanos, 2010).

Accessory proteins such as the PAAR-repeat domain protein are also part of most T6SS and have been shown to play a significant role in T6SS-mediated bacterial competition in *A. baylyi* (Shneider et al., 2013). The PAAR-repeat domain was found to dock onto the terminus of the pilus-like structure formed by VgrG and have been shown to interact with VgrGs and effector proteins, thus functioning as adaptors (Bondage et al., 2016).

The PAAR_like domain superfamily (cl21497; NCBI conserved domains database, CDD) includes proteins bearing PAAR motifs (pfam05488) or the DUF4150 domain (pfam13665). Tandemly repeated PAAR motifs constitute a PAAR domain (cd14671), which has been subclassified in 8 subgroups (cd14737–cd14744), according to the presence of additional N- and C-terminal domains with various predicted functions. Shneider et al. (2013) have classified PAAR-domain containing proteins in 7 classes according to their domain architecture. Following this scheme, Eijkelkamp et al. (2014) have previously detected the presence of PAAR proteins belonging to classes 1 (PAAR-1, N-terminal PAAR domain and no additional domains), 2 (PAAR-2, N-terminal PAAR domain and C-terminal extension of variable length) and 5 (PAAR-5, central PAAR domain) in a group of 8 *A. baumannii* strains (Figure 2B). As described for VgrG proteins, some PAAR proteins have evolved to contain virulence activity in their C-termini (“evolved PAAR”; Shneider et al., 2013).

T6SS involvement in eliminating competitors has been reported in a number of *Acinetobacter* species. *A. nosocomialis* M2 and *A. baylyi* ADP1 were found to utilize the T6SS for dueling against *Escherichia coli* (Carruthers et al., 2013; Shneider et al., 2013). In particular, *A. baylyi* ADP1 encodes 5 different effectors that provoke different degrees of *E. coli* cells lysis (Ringel et al., 2017), namely a putative metallopeptidase (Tpe1), a peptidoglycan-hydrolyzing amidase (Tae1), a phospholipase (Tle1), and two effectors (Tse1, and Tse2) representing new classes of effectors for which no enzymatic activity could be predicted or deduced from the lysis phenotype. Subsequent prey-DNA uptake has been detected, providing a competitive advantage to those strains expressing the T6SS (Ringel et al., 2017), and opening the possibility for the acquisition, among others, of antimicrobial resistance genes (Cooper et al., 2017). Moreover, *A. baumannii* strains DSM30011, ATCC17978 and AB307-0294 were able to outcompete other *A. baumannii* species as well as *E. coli* and *P. aeruginosa*, a bacterial pathogen relevant in mixed nosocomial infections (Weber et al., 2013; Repizo et al., 2015; Fitzsimons et al., 2018). Recently, toxins acting as peptidoglycan hydrolases (LysM), amidases (Tse4), nucleases (Rhs2 and Tse2), lipases (Tse1) and two with unknown function (Tse3 and Rhs1) have been characterized in *A. baumannii* (Weber et al., 2016; Fitzsimons et al., 2018), and many other effectors predicted using bioinformatics (Repizo et al., 2015; Fitzsimons et al., 2018), suggesting these microorganisms possess a significant arsenal of secreted toxins and immunity proteins.

In addition to an established role of the T6SS in bacterial competition, that could be advantageous in *Acinetobacter* colonization of specific environments such as the hospital setting, for some strains the T6SS seems to participate in host colonization, according to assays performed with the insect infection model *Galleria mellonella* (Gebhardt et al., 2015; Repizo et al., 2015). A recent report suggests that the presence of a functional T6SS contributes to infections in immune-compromised patients and those with implanted medical devices (Kim et al., 2017). In this work, the prevalence of the T6SS on 162 *A. baumannii* clinical isolates obtained from patients with bacteremia was also analyzed (Kim et al., 2017). The *hcp* gene was detected in 31.5% of the isolates and its presence showed a clear affiliation to particular STs. This observation is in agreement with genomic comparative analysis of phylogenetically- and epidemiologically-related *A. baumannii* MDR strains showing that the T6MC is only present in particular populations (Wright et al., 2014; Jones et al., 2015). Also, it suggests that this system is not critical in the conditions prevailing in the nosocomial environment. Indeed, the T6SS in *A. baumannii* is clearly tightly regulated, often repressed to promote conjugation and dissemination of MDR-carrying plasmids within a bacterial population (Di Venanzio et al., 2019).

In this study, we aimed at extending the present knowledge on T6SS core and accessory genes and toxins in the *Acinetobacter* genus. Therefore, we carried out an in-depth *in silico* comparative analysis of these clusters in different *Acinetobacter* species to better understand the composition and distribution of T6SS loci in this genus.

## Materials and Methods

### *In silico* Identification of the T6SS Loci

We conducted a comparative genomic analysis including *Acinetobacter* strains available the NCBI-GenBank database. Those strains with a complete genome sequence were included in this analysis. In order to have a full representation of the genus, for those *Acinetobacter* species with no complete genome we decided to work with draft genomes (Supplementary Table S1). We then extracted genomic and proteomic data corresponding to 191 strains and a local database was constructed. Of note, 110 genomes corresponded to *A. baumannii* strains and 81 to non-*baumannii* species, totalizing 42 accepted species and 9 strains so far not assigned to any known species. The proteins encoded within the T6MC from *A. baumannii* DSM30011 (Table 1) which carries a functional T6SS (Repizo et al., 2015) were used as query to perform BlastP-sequence similarity searches (Altschul et al., 1990) against the local database. With these data the corresponding T6SS loci were identified. Furthermore, each of the clusters was manually inspected to corroborate its genetic integrity, and those strains showing incomplete/non-functional clusters were accordingly informed (Supplementary Table S1). Visual representation of the alignments using nucleotide similarities (tblastx) of the T6SS loci were carried out with MultiGeneBlast (default parameters; Medema et al., 2013). Calculations of GC content^1^ and Codon adaptation index (CAI; coRdon) were performed in order to infer if studied regions were acquired by horizontal gene transfer. The CAI of each T6SS gene was normalized using the average CAI of all genes encoded in the corresponding genome. These values were compared with the CAIs obtained for those genes encoding ribosomal proteins in each case, generally accepted as highly adapted to the set of codons available in a particular genome. Statistical analysis (unpaired *t*-test) indicated for each of these genomes a significant difference between both data sets (*p* < 0.0001).

### VgrG, PAAR, and Toxin-Proteins Search

*A. baumannii* DSM30011 encodes 4 VgrG proteins of variable length. However, they all share the basic domain architecture, with a conserved N-terminal VgrG domain. Therefore, the VgrG domain of the protein encoded by DSM30011_13325 (amino acids 23–631) was used as query for a bioinformatic search of homologous proteins in *Acinetobacter* strains contained in our local database. Regarding PAAR-proteins, a homology search was performed using as queries the PAAR-1 and PAAR-2 encoded by *A. baumannii* DSM30011 (DSM30011_11445 and DSM30011_14800), and the PAAR-5 encoded by *A. baylyi* ADP1 (ACIAD0051). Besides, representative proteins found at the NCBI-CDD were used as query in a BlastP-homology search for DUF4150-domain containing proteins. Finally, proteins acting as putative toxins in the *vgrG* and PAAR gene islands were searched with BlastP (*e* < 0.0001), using as query T6SS-reported toxins identified in *Acinetobacter* spp. (Weber et al., 2016; Ringel et al., 2017; Fitzsimons et al., 2018) and in other bacteria (Lien and Lai, 2017; Ma et al., 2018), both targeting prokaryotic and eukaryotic cells.

### Annotation of Proteins With Unknown Function

A number of bioinformatic analyses were conducted in order to infer the role of T6SS-main components and proteins encoded in the *hcp* and *vgrG* islands with unknown function. A search for conserved domains was done by means of the Batch Web-CD search tool (Marchler-Bauer et al., 2015). SignalP 4.0 (Petersen et al., 2011) and TMHMM Server v.2.0^2^ were used to predict signal peptides and trans-membrane helices, respectively.

### Analysis of Genetic Context of PAAR and *vgrG* Islands

In order to analyze the genetic context of the PAAR (not located within T6MCs) and *vgrG* genes, the accession numbers of the 5 downstream and upstream genes were parsed using an R script designed *ad hoc* and the feature table files of the strains under analysis. Then, a search for conserved domains carried by proteins encoded in the PAAR and *vgrG* islands was done using the Batch Web-CD search tool (Marchler-Bauer et al., 2015), and each of them was assigned to a superfamily. Then, the presence or absence of a particular domain superfamily was used as a binary score (present = 1, absent = 0) to perform a hierarchical clustering of PAAR or *vgrG* islands using “pvcluster” R package (Suzuki and Shimodaira, 2006), with binary distance and average agglomerative clustering. Only genetic islands encoding proteins with conserved superfamily domains found at least 3 times in our database were considered. Clustering reliability was assessed by bootstrapping with 1,000 repetitions. The resulting dendrogram was displayed using iTOL (Letunic and Bork, 2011). Those gene islands sharing a similar genetic context were grouped in PAAR or VgrG gene neighborhoods (PGNs and VGNs, respectively).

## Results and Discussion

### T6SS Activity in *Acinetobacter* Strains

T6SS activity has been previously explored in a limited number of *Acinetobacter* spp. (see Introduction). In order to further investigate its function in a larger set of strains, a total number of 20 strains (Table 2) that were available in our laboratory collection were grown and tested. The ability of each *Acinetobacter* strain to eliminate *E. coli* was used as a proxy for an operative T6SS, as previously reported (Carruthers et al., 2013; Shneider et al., 2013; Weber et al., 2013; Repizo et al., 2015; Fitzsimons et al., 2018). Obtained results indicated that 11 out of 20 strains were able to outcompete *E. coli* (Table 2), of which *A. guillouiae* and *A. oleivorans* have never been reported as T6SS-positive strains. T6SS involvement in the observed killing was corroborated by Hcp-secretion assays (Supplementary Figure S1). In view of the observed variability in the killing capacity of different *Acinetobacter* spp. we decided to conduct a bioinformatic search that could shed light on the observed differences.

**TABLE 2:** *Acinetobacter* strains tested for T6SS activity.

**Strain**	**Killing (fold change)^a^**	**Hcp secretion^b^**
*Acinetobacter baumannii* DSM 30011	Complete	+
*Acinetobacter bereziniae* HPC 229	Partial (1.7)	NA
*Acinetobacter bereziniae* LMG 1003	None	NA
*Acinetobacter brisouii* ANC 4119	None	NA
*Acinetobacter calcoaceticus* NIPH 2245	None	NA
*Acinetobacter guillouiae* LMG 988	Partial (3.3)	+
*Acinetobacter gyllenbergii* LUH 4712	Partial (1.9)	NA
*Acinetobacter indicus* ANC 4215	Complete	–
*Acinetobacter johnsonii* DSM 6963	Partial (1.6)	NA
*Acinetobacter junii* SH 205	None	NA
*Acinetobacter lwoffii* DSM 2403	None	NA
*Acinetobacter lwoffii* LMG 985	Partial (7.4)	–
*Acinetobacter nosocomialis* M2	Complete	+
*Acinetobacter nosocomialis* NIPH 2119	Complete	–
*Acinetobacter oleivorans* DR1	Complete	+
*Acinetobacter pittii* LMG 1035	None	NA
*Acinetobacter schindleri* NIPH 1034	None	NA
*Acinetobacter ursingii* NIPH 137	None	NA
*Acinetobacter venetianus* LMG19082	Partial (1.6)	NA
*Acinetobacter* sp. Ver3	None	NA

**^*a*^Killing was calculated by dividing the recovered CFU/ml of E. coli used as prey with respect to a control culture not mixed with any other Acinetobacter strain (see Repizo et al., 2015 for experimental details). ^*b*^Only strains showing killing-fold changes > 2 were analyzed.* NA: not assayed.*

### Identification and Prevalence of Orthologous T6SS Loci in the *Acinetobacter* Genus

Bioinformatic analysis exerted on a local database including 191 *Acinetobacter* genomes (Supplementary Table S1) revealed the presence of genes encoding a T6SS in most *Acinetobacter* species (33 out of 42; Table 3). In some cases, such as *A. lwoffi* and *A. haemol*yticus, we were not able to detect a T6SS loci for those strains included in our local database. However, when performing a broader search against the complete NCBI-Protein database, some strains encoded a T6MC. Therefore, we decided to include these species in Table 3 as well.

**TABLE 3:** Classification of T6SS main clusters present in the *Acinetobacter* genus^a^.

**T6SS**	**Subclass**	**Type**	**Species^b^**
1	A		*A. baumannii, A. calcoaceticus, A. lactucae, A. nosocomialis, A. oleivorans, A. pitti, A. seifertii*
	B	a	*A. baylyi, A. beijerinckii, A. bereziniae, A. colistinresistens, A. equi, A. guillouiae, A. gyllenbergii, A. haemolyticus, A. junii, A. larvae, A. lwoffii, A. proteolyticus, A. radioresistens, A. soli, Acinetobacter* sp. TGL-Y2
		b	*A. apis, A. bohemicus, A. bouvetii, A. celticus, A. gerneri, A. indicus, A. johnsonii, A. pragensis, A. schindleri, A. tandoii, Acinetobacter* sp. LOGeW2-3, ACNIH1
2			*A. gerneri, A. populi, A. rudis*
Negative			*A. brisouii, A. defluvii, A. marinus, A. nectaris, A. parvus, A. puyangensis, A. qingfengensis, A. ursingii, A. twoneri, Acinetobacter* sp. ACNIH2, DUT-2, Ncu2D-2, SWBY1, TTH0-4, and WCHA45

**^*a*^A given species was included in this classification when at least one of its strains contained a complete T6SS loci. ^*b*^Strains correspond to those present in Supplementary Table S1, with the exception of particular cases hereafter indicated. A. haemolyticus strains HW-2A (NZ_CP030880.1) and KCRI-348C (NZ_OVCN01000011.1); A. lwoffi NCTC 5866 = CIP 64.10 = NIPH 512 (APQS01000022.1), NIPH 478 (APQU01000010.1).**

It is worth noting the particular case of *A. towneri* DSM 14962 = CIP 107472, the only strain of this species which genome is available at the non-redundant protein database (NCBI). It encodes a complete T6SS-main cluster, but the *tssH* gene is interrupted by an insertion sequence (IS). No orphan copy of the *tssH* gene was detected, and thus *A. towneri* was thereby considered as a T6SS-deficient strain. Sequencing of other strains is needed to determine if this is the rule for this species. A number of *Acinetobacter* species (8 out of 42), namely *A. brisouii, A. defluvii, A. marinus, A. nectaris, A. parvus, A. puyangensis, A. qingfengensis*, and *A. ursingii* do not encode a T6MC (Supplementary Table S1). Lack of T6SS main genes was also observed for 6 out of 9 *Acinetobacter* spp. (ACNIH2, DUT-2, Ncu2D-2, SWBY1, TTH0-4, and WCHA45).

Regarding the prevalence of the T6MCs in *A. baumannii*, 27 out of 110 strains showed a complete deletion of the T6SS-main cluster, while 15 contained gene frameshifts, partial deletions or insertions resulting in an abnormal T6MC (Supplementary Table S1), very likely conducting to a non-functional apparatus. T6SS genes loss has been already documented in MDR *A. baumannii* clinical strains (Wright et al., 2014; Jones et al., 2015; Kim et al., 2017), suggesting that this system is not critical for survival in the nosocomial environment. Possible hypotheses favoring this genetic loss are higher chances of evasion from the host immune system and/or the lower requirement for interbacterial competition over the course of antibiotic therapy (Repizo, 2017). Furthermore, silencing of the T6SS was recently shown to be critical for horizontal gene transfer through conjugation, which is crucial for antimicrobial resistance spread (Di Venanzio et al., 2019).

### Classification of the T6SS-Main Clusters in *Acinetobacter* Species

Bioinformatic analyses revealed the presence of two types of T6MC in *Acinetobacter* species, here designated as T6SS-1 and T6SS-2 (Figure 1 and Supplementary Figure S2, and Tables 1, 4). Different bacterial species encoding more than one T6MC have been previously reported (Boyer et al., 2009). These two loci differ in genetic composition but both encode the essential components of the T6SS apparatus. The TSS6-1 is the more ubiquitous T6MC in the *Acinetobacter* genus (30 out of 32 T6SS-proficient species; Table 3) and its gene composition is coincident with that reported for *A. baumannii* by Boyer et al. (2009). We established that the main characteristics that define the *Acinetobacter* T6SS-1 are: (1) No *hcp* genes are encoded outside the core cluster; (2) None of its genes code for evolved-Hcp proteins, as previously reported in other bacterial species (Blondel et al., 2009; Ma et al., 2017a); (3) no *vgrG* genes are encoded within the main cluster, in contrast to many other bacteria (Boyer et al., 2009).

**TABLE 4:** T6SS-2-main locus genes in *Acinetobacter* spp.

***A. garneri* DSM14967**						
**Protein^a^**	**COG**	**Locus**	**Length (AA)**	**% AA Id/Sim with *A. rudis* CIP 110305**	**% AA Id/Sim with *A. populi* PBJ7**	**% AA Id/Sim with *B. thailandensis* E264^b,c^**
**TssJ**	3521	F960_01894	218	60/82	50//73	34/56 (41)
**TssK**	3522	F960_01893	446	70/85	60/81	36/54 (99)
**TssL**	3455	F960_01892	415	77/87	71/81	37/59 (90)
**TssM**	3523	F960_01891	1176	69/83	61/78	25/45 (99)
TagF	3913	F960_01890	182	58/78	38/63	24/35 (48)
**TssA**	3515	F960_01889	349	62/80	59/76	34/52 (96)
**TssB**	3516	F960_01888	170	85/93	81/94	65/83 (90)
**TssC**	3517	F960_01887	494	93/98	89/96	72/87 (96)
**TssD/Hcp**	3157	F960_01886	161	95/98	88/97	48/66 (97)
ImpE	4455	F960_01885	255	49/65	38/62	–
**TssE**	3518	F960_01884	159	74/86	67/82	33/57 (91)
**TssF**	3519	F960_01883	623	70/86	58/76	24/43 (99)
**TssG**	3520	F960_01882	328	70/85	60/79	– (Pseudogene)
**TssH/ClpV**	0542	F960_01881	879	80/89	72/85	53/71 (99)
PAAR	4104	F960_01880	88	82/92	52/74	–
**VgrG**	3501	F960_01907	894	29/47		37/55 (61)

**^*a*^Core components described by Boyer et al. (2009) are shown in bold. ^*b*^Sequence homology with proteins encoded in the T6SS-6. ^*c*^Query coverage is indicated in parenthesis.**

A search for T6SS-1-core proteins in non-*Acinetobacter* species, using as query *A. baumannii* DSM30011 conserved components (TssB, TssC, TssK, TssL) indicated the presence of homologous proteins (identity > 45%) in members of several genera, namely *Alkanindiges, Psychrobacter, Achromobacter, Chitinimonas*, and *Cupriavidus*, and as previously noticed with those which are frequently associated to plants such as *Ralstonia, Burkholderia*, and *Xanthomonas* (Boyer et al., 2009; Spiewak et al., 2019). However, it is important to notice that the genetic architecture of T6SS-core clusters in these species is diverse. It has already been demonstrated that the T6SS mediates bacterial interactions with host plants, through the secretion of effectors which act in symbiosis, biofilm formation, virulence, and interbacterial competition (Ryu, 2015). Given the fact that several *Acinetobacter* species have been isolated from plants, including the enviromental isolate *A. baumannii* DSM30011 (Repizo et al., 2017), it is not surprising that they might have originally acquired T6SS-core genes from other plant associated bacteria and subsequently modeled these clusters to the actual genetic organization.

The T6SS loci encoded by most bacteria are organized in operons, suggesting a coregulated expression (Bernard et al., 2010). Therefore, we investigated the presence of putative transcriptional units in the *A. baumannii* T6SS-1 main cluster. According to bioinformatic predictions available at ProOpDb (Taboada et al., 2012), two transcriptional blocks might be present in these loci, the first encompassing *hyp1*-PAAR genes and the second including *tssH-tagX* genes (Figure 1A). The separation of this locus into two transcriptional units might explain the existence of two different genetic arrangements among T6SS-1 gene clusters (Figure 1A):

(i) T6SS-1A: In this case, all the T6MC genes are encoded in the same DNA strand. This genetic architecture is conserved in *Acinetobacter* species belonging or phylogenetically related to the *calcoaceticus-baumannii* complex (Table 3).
(ii) T6SS-1B: In this T6MC the *tssH*-*tagZ* gene block is divergently located to the *hyp1*-PAAR gene block (Figure 1A). This is the more ubiquitous organization (23 out of 32 species; Table 3) and for clarity, we further classified it in two types depending on the presence (T6SS-1Ba) or not (T6SS-1Bb) of the gene encoding a PAAR domain containing-protein within the locus. In line with this observation, the PAAR domain-protein encoded in the main cluster is not essential for T6SS functioning in *A. baylyi* (Weber et al., 2016). This is not surprising since more than one gene is usually present per *Acinetobacter* genome (see below).

We observed a strain clustering that was in line with the proposed sub-classification (Supplementary Figure S2 heat map), with some exceptions mostly corresponding to ACB complex species carrying T6SS-1A loci that did not group with the larger block of T6SS-1A ACB strains. This might be an indication of gene acquisition by horizontal gene transfer. Furthermore, ST affiliation of each *A. baumannii* strain was included in this same figure, showing a sub-clustering, which correlated with the ST classification (Pasteur Scheme).

The role played by three of the genes present in the *Acinetobacter* T6SS-1 encoding cluster is still unknown (ACIAD2693, ACIAD2685, and ACIAD2698), although deletion of any of the former two, coding for hypothetical proteins 1 (Hyp1) and 2 (Hyp2), affects the functionality of the system (Weber et al., 2016; Ringel et al., 2017). We therefore decided to investigate if we could infer putative functions based on bioinformatic predictions carried out with the DSM30011 homologous proteins. We observed that Hyp1 (DMS30011_11500) bears no putative transmembrane segments and was predicted to carry a signal peptide which might direct it to the periplasm (Supplementary Figure S3A). Interestingly, we were able to identify in DSM30011 Hyp1 several features of its signal peptide sequence, which are characteristic of lipoproteins. It possesses a C23 residue which is part of a lipobox motif (PROSITE pattern PS51257), a positively charged K5 residue and a stretch of hydrophobic and uncharged amino acids residues between position 7 and 22 (Zückert, 2014). These structural characteristics resemble those of TssJ, which has been proposed to anchor the membrane complex (MC) to the inner membrane (IM) through its interaction with TssM (Felisberto-Rodrigues et al., 2011). Moreover, TssJ bears a lipobox motif which is critical for the association of the mature protein with the outer membrane through acylation of the N-terminal cysteine residue (Aschtgen et al., 2008). However, no protein showing homology with TssJ could be identified (Weber et al., 2013). Therefore, we propose that Hyp1 encoded within the *A. baumannii* T6MC could fulfill a role similar to that played by TssJ.

In enteroaggregative *E. coli* and in several other bacteria, TagL associates with the TssJLM membrane complex (Aschtgen et al., 2010). TagL is embedded in the IM through 3 transmembrane regions. It also bears a central cytoplasmic loop and a C-terminal periplasmic domain with a functional peptidoglycan-binding motif (Aschtgen et al., 2010), which tethers the T6SS to the cell wall. The peptidoglycan binding motif may also be encoded in a separate subunit, TagN. We and others (Ringel et al., 2017) observed that this is the case in *Acinetobacter* species. *A. baumannii* DSM300011 TagN bears an OmpA_C-like peptidoglycan binding-domain (152–254; cd07185; Supplementary Figure S4A) and a putative exportation signal (cleavage site between positions 27 and 28: ALA-QP). The obvious question is which *Acinetobacter* protein fulfills the role played by the N-terminal portion of *E. coli* TagL. Topology predictions performed on Hyp2 (DSM30011_11465) indicates that this could be a good candidate to play this role, since it contains two putative transmembrane domains in the N-terminal followed by a long periplasmic region (Supplementary Figure S3B). Recent predictions consistent with this hypothesis have been made when analyzing the topology of a homologous protein (TagZ) encoded by *Burkholderia*, *Paraburkholderia* and related species (Spiewak et al., 2019), which share a T6SS-1 main cluster with a similar gene composition but with a different arrangement with respect to *Acinetobacter* spp.

Hyp3 (DSM30011_11420) contains a C-terminal domain (PRK03427) found in the *E. coli* cell division protein ZipA, which is tied to the membrane through a short N-terminal membrane-anchored domain. Correspondingly, Hyp3 bears a putative transmembrane domain in its N-terminal region (residues 21–43; Supplementary Figure S3C) with the rest of the protein protruding toward the periplasm. Despite the fact that this protein shows low conservation in the *Acinetobacter* genus, its predicted topology is conserved. Weber et al. (2016) have suggested that TagX interaction with other components of the T6SS apparatus may control its enzymatic activity, allowing for precise spatial regulation of PG degradation. It is therefore possible that Hyp3 fulfils this role but experimental work needs to be carried out to test this hypothesis.

### VgrG Protein Diversity in *Acinetobacter* spp.

Varying numbers of genes located outside the *Acinetobacter* T6MC encode putative VgrG proteins (Weber et al., 2013). These T6SS-associated regions additionally encode a variable number of accessory and hypothetical proteins that act as effectors and immunity proteins (see Toxins section). VgrG proteins encoded in *Acinetobacter* genomes under study were investigated (see section Materials and Methods). As result, 534 homologous proteins were detected (average *vgrG* genes per genome = 2.79; Supplementary Table S2) throughout the *Acinetobacter* genus. In correlation with the lack of T6SS structural components, 7 out of 8 T6SS- *Acinetobacter* species (with *A. ursingii* as the exception; Table 3) do not encode *vgrG* genes. Lack of *vgrG* genes was also observed for the aforementioned T6SS-deficient *Acinetobacter* sp. Ncu2D-2, TTH0-4 and WCHA45 (Supplementary Table S1). Interestingly, species such as *A. celticus*, *A. equi*, *A. pragensis*, and *A. radioresistens* encode only 1 *vgrG* gene, whereas *A. bereziniae* carries 13 (Supplementary Table S2). *A. baumannii* T6SS-proficient strains usually contain between 2 and 4 copies. Interestingly, some *Acinetobacter* strains, show a split *vgrG* locus encoding separate N-terminal and C-terminal VgrG domains. This split among VgrG domains resembled the case of the bacteriophage spike proteins, and supported the proposed common evolutionary origin of these two membrane-penetrating devices (Pukatzki et al., 2007; Zoued et al., 2014). In addition, 3 of the identified VgrG sequences in our database contain putative effector domains in their C-termini (Supplementary Tables S2, S3). These evolved-VgrGs, which were identified in *A. bereziniae* and *A. proteolyticus*, encode both LysM and Het-C domains (Table 5). While the former (cd00118) is responsible for protein binding to peptidoglycan (Buist et al., 2008), the latter (pfam07217, cl20332 superfamily) is found in proteins regulating self/non-self-recognition in filamentous fungi (Wu et al., 1998). Antifungal effectors dependent on the T6SS have been recently described in *Serratia marcescens* (Trunk et al., 2018), therefore it is possible that those *Acinetobacter* VgrG proteins bearing a Het-C domain fulfill a similar function. In sum, these predictions suggest that some of these VgrGs may have additional functions, adding further levels of complexity to the *Acinetobacter* T6SS toxins repertoire.

**TABLE 5:** VgrG-evolved proteins in *A. bereziniae*.

**VgrG-evolved proteins in *Acinetobacter* species**						
**Organism**	**Protein accession no.**	**Length (AA)**	**Start**	**End**	**Extra domain^a^**	
					**Domain**	**Domain accession no.**
*A. bereziniae*	ATZ63530.1	1738	995	1037	LysM	cd00118
*A. bereziniae*	ATZ63530.1	1738	1320	1688	Het-C superfamily	c120332
*A. bereziniae*	ATZ63848.1	1737	991	1034	LysM	cd00118
*A. bereziniae*	ATZ63848.1	1737	1313	1678	Het-C superfamily	c120332

*^*a*^Obtained from the NCBI-CDD (Marchler-Bauer et al., 2015).*

### The Newly Identified T6SS-2 Is Present in Three Environmental *Acinetobacter* Species

As previously mentioned, a T6MC with a different gene composition (T6SS-2) was also detected. We identified the T6SS-2 locus in *A. gerneri* DSM 14967 (also encoding a T6SS-1Bb cluster), isolated from activated sludge (Carr et al., 2003); in *A. rudis* CIP 110305 isolated from raw milk, and in *A. populi* PBJ7, isolated from a bark canker of *Populus euramericana* (Table 4 and Supplementary Table S1). It is composed of 15 genes, including the 13 T6SS-core genes described by Boyer et al. (2009); Figure 1B and Table 4). It shares 13 orthologous genes with the T6SS-1 main cluster, with *tssJ* and *impE* as genes specific of the T6SS-2 cluster. Key components (TssB, TssC, TssK, TssL) are highly conserved (aminoacidic identity > 60%) among the three aforementioned *Acinetobacter* species (Table 4). Remarkably, the PAAR gene is encoded in the complementary strand in *A. gerneri* and *A. rudis*, whereas in *A. populi* it is located in the same strand as the rest of the cluster. This observation is in line with the documented variability of PAAR genes location in the *Acinetobacter* T6SS-1 core clusters.

T6SS-2 organization resembles that of the gene cluster encoding the T6SS-6 described in *Burkholderia* species (Figure 1B and Table 4; Schwarz et al., 2014) with genes encoding VgrG proteins, putative toxins and cognate immunity proteins all located in the proximity of the main cluster in these three *Acinetobacter* strains. Still, important differences in genetic composition of the main cluster are observed between them such as the presence of the *impE* gene in the *Acinetobacter* T6SS-2, which is part of the T6MC in *Rhizobium leguminosarum* (Bladergroen et al., 2003), and the absence of the *ppkA* gene, encoding a kinase involved in post-translational regulation of the system in *P. aeruginosa* (Basler et al., 2013). Interestingly, the *A. gerneri* T6SS-2 specific components (meaning those for which no homologous proteins are encoded in the T6SS-1 cluster also encoded in its genome), namely TssJ, TssL and ImpE, show the highest identity percentages (between 35 and 50%) with proteins present in members of the *Moraxella* and *Neisseria* genera, which carry an unreported T6SS cluster with a different genetic structure. Analysis of G + C% of the T6SS-2 region in each of the three above mentioned *Acinetobacter* strains shows a significant deviation from the genomic average (35.8% vs. 37.9% for *A. gerneri* DSM14967; 33.3% vs. 39.2% for *A. rudis* CIP110305; and 32.6% vs. 40.2% for *A. populi* PBJ7). Moreover, average codon adaptation index calculations for each of these T6SS-2 clusters indicate a significant deviation (*p* < 0.0001) from the results obtained with the corresponding ribosomal proteins of each organism. Overall, these predictions imply a recent acquisition of this cluster by horizontal gene transfer from an unknown bacterial donor, thereby suggesting that the T6SS-1 and T6SS-2 clusters are evolutionarily distinct.

Remarkably, *Acinetobacter* species carrying the T6SS-2-core cluster encode several VgrG proteins with an additional domain (T6SS_Vgr; Supplementary Table S3) not frequently detected in VgrG present in other members of this genus (Figure 2A). The T6SS_Vgr domain (pfam13296) overlaps with the C-terminal part of the VgrG domain and is usually located before the start of the DUF2345 domain. Of note, *A. populi*, which is the only *Acinetobacter* species under study carrying uniquely a T6SS-2 main cluster, solely encodes VgrG proteins (total number = 3) with this domain architecture in its genome (Supplementary Table S2). On the other hand, *A. gerneri* (T6SS-1 and -2 clusters; total number = 9) and *A. rudis* (incomplete T6SS-1 and complete T6SS-2 clusters; total number = 8) inspected strains encode VgrG proteins falling into both domains architectures (Figure 2A and Supplementary Tables S2, S3). Furthermore, we detected that these types of VgrGs are encoded by genes which are usually in close proximity to the T6SS-2 main cluster. It is tempting to speculate that VgrGs bearing the T6SS_Vgr domain are somehow related to the apparatus encoded by T6SS-2-main cluster. An additional evidence supporting this hypothesis is that the most similar VgrG proteins outside the *Acinetobacter* genus are encoded by members of the *Moraxella* genus (40% average identity), in agreement with what has been observed for proteins encoded in the T6SS-2 main cluster (see above). This suggests that the complete system (T6MC plus VgrG proteins) has been acquired from species of the *Moraxella* genus.

Overall, these observations suggest that the *Acinetobacter* T6SS-2 cluster has a unique genetic organization and composition so far not described in other bacteria. Future work needs to be directed toward understanding the role that this T6SS plays in the biology of these *Acinetobacter* spp.

### Three Major Classes of PAAR Domain Containing-Proteins Are Found in *Acinetobacter* spp.

A bioinformatic search for PAAR proteins in members of the *Acinetobacter* genus was conducted (see section Materials and Methods). As result, 377 homologous proteins were detected (average PAAR genes per genome = 1.97; Supplementary Table S4) throughout the *Acinetobacter* genus. An interesting case is that of *A. baumannii* SDF which carries 10 PAAR genes (Eijkelkamp et al., 2014), namely 1 PAAR-1, 7 PAAR-2, and 2 PAAR-5. Remarkably, one of the PAAR-2 proteins (CAP02972) is encoded in a plasmid (p2ABSDF, CU468232) as part of a locus carrying two *vgrG* genes and several putative toxins, such as a potential amidase (CAP02973). Plasmid-borne T6SS genes have been described in other bacteria such as *Pantoea ananatis* (Shyntum et al., 2014), but so far not in *Acinetobacter* species. On the other hand, 7 out of 8 T6SS^–^ *Acinetobacter* species (with *A. ursingii* as the exception; Table 3) do not encode any PAAR gene, in correlation with the lack of the structural components of the T6SS. Lack of PAAR genes was also observed for all the aforementioned T6SS^–^ *Acinetobacter* members still not assigned to any species (Supplementary Table S1).

By means of this analysis we also determined that the majority of *Acinetobacter* PAAR proteins (Figure 2B and Table 6) belong to Shneider’s classes 1 (PAAR_CT_2 domain, cd14744 of NCBI-CDD), 2 (PAAR_CT_2 domain or PAAR_motif), and 5 (PAAR_motif; Supplementary Table S5). Interestingly, the PAAR proteins encoded within the T6SS-main clusters all belong to class 1, with the exception of *A. colistinresistens* NIPH 1859 which encodes a PAAR-2 protein (ENX34299) of 176 amino acids (Table 5 and Supplementary Table S4) and no other PAAR-1 elsewhere in its genome. Through this analysis we also observed that all *Acinetobacter* species bearing a T6SS-1Bb cluster (lacking a PAAR gene in the main cluster), encode an orphan PAAR-1 elsewhere in the genome. With the exception of *A. indicus* SGAir0564 (3 PAAR genes in total) and *A. gerneri* DSM 14967 = CIP 107464 (6 PAAR genes in total; also encoding a T6SS-2 main cluster) this is the only PAAR gene copy they carry in their genomes. This suggests that the PAAR-1 proteins are the T6SS-apparatus dedicated proteins in *Acinetobacter* species.

**TABLE 6:** PAAR-domain proteins in *Acinetobacter* spp.

**Classification of PAAR-domain proteins in the *Acinetobacter* genus**				
**Organism**	**Location**	**Length (AA)**	**Classification^a^**	**Occurrence**
Several species (T6SS-1A, T6SS-1Ba, T6SS-2)	Main cluster	86–88	1	119
Several species (T6SS-1Bb)	Orphan PAAR gene	86–88	1	16
*A. colistiniresistens* NIPH 1859	T6SS-1Ba (main cluster)	176	2a	1
Several species	Orphan PAAR gene	96–368	2a	177
Several species	Orphan PAAR gene	171–399	2b	9
*A. baumannii, A. oleivorans*	Orphan PAAR gene	125–257	2c	26
Several species	Orphan PAAR gene	133–161	5	29

**PAAR-2b proteins in the *Acinetobacter* genus**				
**Organism**	**Accession number**	**Length (AA)**	**Extra domain^b^**	

*A. lactucae* OTEC-02	ARD27797	178	DUF2345 superfamily	
*A. gyllenbergii* NIPH 230	ESK35400	172	DUF2345 superfamily	
*A. indicus SGAir0564*	AVH13769	284	Pox_polyA_pol_C superfamily	
*A. gyllenbergii* FMP01	OBY73915	171	SdrD_B superfamily	
*A. proteolyticus 2P01AA*	PKF32710	171	SdrD_B superfamily	
*A. guillouiae* NIPH 991	ENV17008	183	DUF2345 superfamily	
*A. colistiniresistens* NIPH 2036	EPG34566	181	HP superfamily	
*A. guillouiae* CIP 63.46	ENU60235	399	Lysozyme_like superfamily	
*A. baumannii* SDF	CAP02689	183	YbbN superfamily	

**^*a*^Adapted from Shneider et al. (2013). ^*b*^Obtained from the NCBI-CDD (Marchler-Bauer et al., 2015).**

We also noticed that PAAR-2 proteins could be sub-classified into 3 classes (2a to 2c) according to their domain organization (Figure 2B), with those belonging to class 2b bearing additional domains (evolved-PAAR proteins; Table 6). Some of the latter carry a DUF2345 domain (pfam10106), usually part of VgrG proteins, suggesting it could fulfill an adapter role. Others bear domains probably acting as toxins, such as those belonging to the Lysozyme-like superfamily (cl00222). However, only 9 PAAR-2b proteins were detected and according to predictions in most cases the additional domain is not complete (Supplementary Table S5). Another aspect that is worth-commenting is that PAAR-2c proteins were mostly encoded by *A. baumannii* strains (25 out of 26; Supplementary Table S4).

With respect to PAAR-5 proteins, they are the less represented with only 29 members among *Acinetobacter* strains under study (Table 6). This is coincident with previous analysis performed on 1,353 bacterial PAAR proteins (24 occurrences, Shneider et al., 2013). Another aspect that is interesting to mention, is that PAAR-5 genes are usually located in tandem with a PAAR-2 gene (Supplementary Table S4) as has been described for *A. baylyi* ADP1 (CAG67033 and CAG67034; Shneider et al., 2013) and that these regions usually encode toxin genes (see below).

The genetic variability within each PAAR class was lastly investigated, based on the identity percentage of PAAR proteins under study with respect to *A. baumannii* DSM30011 PAAR-1 (Supplementary Table S4). We observed a significant level of conservation among PAAR-1 proteins encoded within the T6MC-1A and 1Ba (%Id 64–100), which was also evidenced from the heatmap (Supplementary Figure S2). These levels of conservation were also detected for 12 out of 15 PAAR-1 proteins present in the genomes of strains carrying the T6MC-1Bb (%Id 66–82; with respect to *Acinetobacter* sp. ACNIH1 PAAR-1). High conservation levels were also observed for PAAR-2c proteins (%Id 83–87), mainly encoded in *A. baumannii* strains. In turn, other groups showed an important variability, such as PAAR-2a (Id% 30–88) and PAAR-5 (Id% 40–100; with respect to *A. baylyi* ADP1 PAAR-5), and as expected PAAR-2b (%Id 31–85), since they carry an extra domain. It is tempting to speculate that those PAAR proteins fulfilling a structural role tend to be more conserved than those probably acting as toxin-chaperones (see below).

### Two Undescribed PAAR-Rhs Proteins Probably Acting as Toxins

Since none of the PAAR proteins identified through this analysis bore a DUF4150 domain, also found in proteins belonging to the PAAR_like domain superfamily, a search for proteins carrying this domain was conducted. By this means, two proteins present in *A. gerneri* DSM 14967 = CIP 107464 (ENV33198) and *A. equi* (ALH95481) with significant homology were detected. These proteins additionally carried RhsA domains (cl27255; Supplementary Figure S4B) and thus could be categorized as PAAR-3 proteins according to a previous classification (Shneider et al., 2013). A predicted toxin domain (pfam15633) of the ADP-ribosyltransferase superfamily was detected in ENV33198. PAAR-3 proteins carrying this toxin domain (categorized as “protein-modifying”) have been identified in 16 bacterial species (Ma et al., 2017b). The genes encoding these PAAR-3 proteins, are preceded by genes coding for a protein carrying a DUF2169 domain and a “short” VgrG (<750 aa, lacking a DUF2345 domain). We found that these clusters are also present in bacteria belonging to diverse genera such as *Bordetella*, *Burkholderia*, *Variovorax*, *Alcalinovorax*, and *Pseudomonas*. Furthermore, a DUF2169 domain-carrying protein fulfill an adapter role specific for the Tde2 toxin (AAK89757; carrying a DUF4150 and a Ntox15 DNase domain) in *Agrobacterium tumefaciens* (Bondage et al., 2016). Interestingly, the gene encoding for the DUF2169-domain–containing protein is always located between the *vgrG2* and *tde2* genes. Conservation in gene cluster organization across diverse Proteobacterial lineages has also been reported. All these data are on favor of the hypothesis that the Tde effector is stabilized and carried by its cognate adaptor/chaperone, which loads the effector onto the C terminus of VgrG for secretion across bacterial membranes (Bondage et al., 2016). It is thus tempting to speculate that the DUF2169 domain-carrying protein present in *Acinetobacter* strains is involved in the loading of PAAR-3 partners.

### T6SS-Associated Toxin Repertoire in *Acinetobacter* spp.

In order to search for putative toxin encoding genes in the proximity of *vgrG* and PAAR identified genes, a homology search using as query T6SS-reported toxins was performed (see section Materials and Methods and Supplementary Table S6). Gene neighborhoods of PAAR and *vgrG* genes were further investigated in order to analyze the existence of specific genomic spots where PAAR and *vgrG* islands are located, the *Acinetobacter* species encoding specific islands, the presence of different classes of PAAR or VgrG proteins linked to these neighborhoods and finally, the toxins associated to each of them. With all this information, a clustering approach was followed in order to distinguish either PAAR or VgrG proteins sharing common genetic environments (Supplementary Figures S5, S6). According to this analysis 6 PAAR- and 14 VgrG-gene neighborhoods (PGN and VGN, respectively) were defined (Supplementary Table S7), which are summarized in Table 7. We observed that a gene encoding for a SDR family NAD(P)-dependent oxidoreductase is part of several of these gene neighborhoods (PGN5, PGN6, and VGN6), suggesting this genome region might be a conserved spot for PAAR and *vgrG* islands. Moreover, 12 out of 15 PAAR-1 genes present in the genomes encoding a T6SS1-Bb-core cluster grouped in PGN4, whereas the remaining 3 (ENV70927, SNQ29620, and ENV33195) were located within mixed PAAR-*vgrG* islands not associated to any particular PGN/VGN (Supplementary Figure S5). The conservation of PGN4 reinforces the idea that the PAAR-1 proteins encoded in these clusters are devoted to the T6SS.

**TABLE 7:** Putative toxins identified in the PAAR and *vgrG* gene neighborhoods^a^.

**PGN^b^**	**Anchor gene function (gene name)**	**PGN occurrence**	**PAAR length range**	**PAAR class**	**Toxin**			
					**Domain**	**Domain superfamily**	**Function**	***Acinetobacter* spp.**
1	Outer membrane porin, OprD family	9	181 (8)	2a (9)				
2	NUDIX hydrolase	9	Variable	2a (9)	T6SS amidase effector protein 4 (6)	cl16625	Amidase	*A. gyllenbergii* (3), *A. colistiniresistens* (2), *A. baumannii* (1), A. *proteolyticus* (1), *A. towneri* (1), *A*. sp. TGL-Y2 (1)
3	Uncharacterized protein (NlpC/P60 superfamily domain)	46	256–280 (42)	2a (41)/2c (5)				
4^c^	Pimeloyl-ACP methyl ester carboxylesterase	12	88 (12)	1 (88)				
5	SDR family NAD(P)-dependent oxidoreductase	61	279 (45)/256 (16)	2a (45)/2c (16)				
6	SDR family NAD(P)-dependent oxidoreductase	6	272 (4)/249 (2)	2a (4)/2c (2)				

**VGN^b^**	**Anchor gene function**	**VGN occurrence**	**VgrG length range**	**Other associated proteins**	**Toxin**			
					**Domain**	**Domain superfamily**	**Function**	***Acinetobacter* spp.**

1 (Tse4^d^)	Disulfide bond formation protein (*dsbB*)	120	1093–1133 (106)		Lysozyme_like (5)	cl00222	Beta-1,4- linked polysaccharides hydrolysis	*A. baumannii* (94), *A. beijerinckii* (2), *A. calcoaceticus* (2), *A. colistiniresistens* (2), *A. gerneri* (1), *A. gyllenbergii* (1), *A. haemolyticus* (1), *A. junii* (1), *A. lactucae* (1), *A. nosocomialis* (4), *A. oleivorans* (1), *A. pittii* (4), *A. populi* (1), *A. proteolyticus* (1), *A. seifertii* (3), *A*. sp. DUT-2
					LysM (30)	cl28306/cl21525	Peptidoglycan binding in bacteria and chitin in eukaryotes	
					Peptidase_M23 (23)	cl27539	Zinc-dependent metallopeptidase (Gly-Gly endopeptidases)	
					LysM + Amidase_5 (12)	cl28306/cl21525 + cl21534 (NlpC/P60)	N-acetylmuramoyl-L-alanine amidase	
					Lysozyme_like + LysM (12)	cl00222 + cl28306/cl21525	see above	
					LysM + Peptidase_M23 (32)	cl28306/cl21525 + cl27539	see above	
2	Patatin-like phospholipase	4	878–879 (4)	DUF4123-containing protein	Metallo-peptidase family M12 (4)	cl00064	Zinc-dependent metalloprotease	*A. guillouiae* (3), *A. bereziniae* (1)
3	Methionine aminotransferase	9	variable		lysozyme_like (1)	cl00222	See above	*A*. sp. (1)
					LysM (3)	cl28306/cl21525	See above	*A. bohemicus* (1), *A. celticus* (1), *A. pragensis* (1)
					LysM + Peptidase_M23 (1)	cl28306/cl21525 + cl27539	See above	*A. schindleri* (1)
4 (Tae1^e^)	Malate dehydrogenase	16	1095–1129 (13)		lysozyme_like (1)	cl00222	see above	*A. soli* (1)
					LysM (4)	cl28306/cl21525	see above	*A. baylyi* (2), *A. radioresistens* (1), *A. guillouiae* (1)
					Peptidase_M23 (1)	cl27539	See above	*A. guillouiae* (1)
					lysozyme_like + LysM (1)	cl00222 + cl21525	See above	*A. ursingii* (1)
					LysM + Peptidase_M23 (2)	cl28306/cl21525 + cl27539	See above	*A. ursingii* (1), *A. johnsonii* (1)
5 (Tse2^e^)	Enoyl-[acyl-carrier-protein] reductase	4	Variable		Undetermined (3)			*A. baylyi* (1), *A. guillouiae* (1)
6	SDR family NAD(P)-dependent oxidoreductase	27	920–948 (27)	Sel1 repeat containing protein (cl27881), DUF3396 (cl13337)	Undetermined (2)		Putative lipase	*A. baumannii* (25), *A. pittii* (1), *A. nosocomialis* (1)
7	Uncharacterized protein (NTF2_lilke superfamily domain)	14	876 (13)	DUF4123-containing protein	Undetermined			*A. baumannii* (12)
8 (Tse3^d^)	T6SS-1a main cluster	5	926–928 (5)		Undetermined		Amidase	*A. pittii* (2), *A. baumannii* (2)
					RhsA + NUC (1)	cl27255/cl04135 + cl00089	DNA/RNA non-specific endonuclease	*A. pittii* (1)
					RhsA + Tox-ART-HYD1 (1)	cl27255/cl04135 + cl21425	Toxin of the ADP-ribosyltransferase superfamily	*A. pittii* (1)
9 (Tse2^d^)	DNA-binding transcriptional regulator, AcrR family	10	94–947 (6)		Ntox15 (6)	cl21405	Predicted RNase toxin	*A. baumannii* (4), *A. pittii* (1), *A. bereziniae* (1)
					DUF2235 (1)	cl01480	Alpha/beta hydrolase	*A. pittii* (1)
10	5-carboxymethyl-2-hydroxymuconate Delta-isomerase	6	Variable	Sel1 repeat containing protein (cl27881)	LysM (2)	cl28306	See above	*A. gyllenbergii* (1), *A. colistiniresistens* (1)
11	LysR-family transciptional regulator	7	875–913 (7)	DUF4123-containing protein	DUF2235 (5)	cl01480	Alpha/beta hydrolase	*A. pittii* (2), *A. baumannii* (1), *A. seifertii* (1)
					Ab_hydrolase (2)	cl21494	Alpha/beta hydrolase	*A. guillouiae* (1), *A. bereziniae* (1)
12 (Rhs-2^f^)	Beta-lactamase class A (*penP*)	88	1048–1060 (84)		RhsA + Tox-GHH (57)	cl27255/cl04135 + cl21428	Toxin of the HNH/Endonuclease VII fold superfamily (sG[HQ]H motif)	*A. baumannii* (74)
					RhsA + AHH (11)	cl27255/cl04135 + cl16862	Nuclease of the HNH/ENDO VII superfamily (AHH motif)	
					RhsA + YwqJ-deaminase (6)	cl27255/cl04135 + cl24268	Toxin of the nucleic acid/nucleotide deaminase superfamily	
					RhsA + undetermined (9)	cl27255/cl04135		
13 (Tse1^d^)	3-oxoacyl-(acyl carrier protein) synthase I	8	918–952 (8)	Sel1 repeat containing protein (cl27881)	Undetermined (5)		Putative lipase	*A. baumannii* (5)
14 (Rhs-1^f^)	DNA-binding transcriptional regulator, AcrR family	67	1039–1164 (61)	Sel1 repeat containing protein (cl27881)	RhsA + YwqJ-deaminase (1)	cl27255/cl04135 + cl24268	See above	*A. tandoii* (1)
					RhsA + Bacuni_01323_like (1)	cl25912	Uncharacterized protein conserved in Bacteroidetes	*A. guillouiae* (1)
					RhsA + undetermined (63)	cl27255/cl04135		*A. baumannii* (58)

**^*a*^Numbers in parenthesis indicate the occurrence of the given feature. ^*b*^Characterized toxins belonging to a specific PGN/VGN are indicated in parenthesis. ^*c*^This PGN nucleates PAAR-proteins present in *Acinetobacter* spp. bearing a T6SS-1Bb main cluster. ^*d*^Weber et al. (2016). ^*e*^Ringel et al. (2017). ^*f*^Fitzsimons et al. (2018).**

In correlation with toxin genes, we initially observed that *vgrG* islands were particularly enriched in genes encoding different classes of previously reported toxins whereas in PAAR islands mostly Tpe1 and Tae4 toxins were detected (Supplementary Table S6). In *A. baylyi* ADP1 the *tpe1* gene is located downstream a PAAR-2 encoding gene (Ringel et al., 2017). In agreement with this data, our analysis detected only 1 *tpe1* orthologous gene next to a *vgrG* gene whereas 20 were in close proximity to a PAAR gene (Supplementary Table S6). Furthermore, we observed that in 11 out of these 20 loci, there is a PAAR-5 gene upstream the PAAR-2 gene (Supplementary Table S6). This information suggests that these toxins are usually genetically linked to PAAR genes in *Acinetobacter* spp. Interestingly, *A. colistineresistens* encodes a Tpe1 toxin (ENX34300) in the T6MC next to an unusual PAAR-2a gene. While Tpe1 proteins are not particularly associated to any PGN, putative Tae4 proteins are encoded in the PGN2 (Supplementary Figure S5). We noticed that 6 out of the 9 clusters included in PGN2 encoded Tae4-amidase effectors accompanied by their immunity proteins (Tai4; Table 7). Of note, the Tae4-Tai4 pair (CAP02973-CAP02974) is encoded in *A. baumannii* SDF p2ABSDF. Furthermore, 5 PAAR genes (ATZ62304, ESK36633, OBY73307, ATZ64949, AMW78666; Supplementary Figure S5) were found to be accompanied by a gene encoding a protein with a VRR_Nuclease domain (cl22959) and followed by 1 to 4 genes bearing a DUF3396 domain (cl13337). These PAAR proteins all belong to class 2a and are between 176 and 179 aa long (Supplementary Table S4). It would be interesting to determine if they are responsible for the secretion of the putative nuclease effector and if the DUF3396-proteins are involved in this task as well.

When analyzing the different VGNs, we observed that VGN1 (the most prevalent), 3, 4, and 10 encoded peptidoglycan hydrolases, M12 and M23 domain peptidases, LysM-domain containing proteins and numerous proteins of unknown function. This is in agreement with a previous report including 23 *Acinetobacter* genomes (Fitzsimons et al., 2018). We also detected the presence of proteins with significant homology with toxins previously reported in *Acinetobacter* spp. (Weber et al., 2016; Ringel et al., 2017; Fitzsimons et al., 2018), associated to specific VGNs (Table 7). Several DUF4123 proteins were found in VGN2, 7 and 11, accompanied by a “short VgrG” (875–883 aa) and associated to Tle1 toxins (DUF2235) or proteins carrying a M12 metallo-peptidase family domain (pfam13688). The presence of DUF4123 proteins in VGNs has already been pinpointed by Fitzsimons et al. (2018). Ringel et al. (2017) identified in *A. baylyi* the Tap-1 chaperone (carrying a DUF4123 domain) necessary for the putative Tse2 toxin delivery. Therefore, it is possible that these proteins might play a role as chaperones for different toxin partners.

In this analysis we were also able to identify a vast repertoire of Rhs-domain containing proteins, which were found to be present in *vgrG* islands of 13 out of the 33 *Acinetobacter* T6SS-proficient species under study (Supplementary Table S6). Moreover, they are clearly conserved in *A. baumannii* strains (90 out of 110). Rhs-encoding genes were particularly grouped in the VGN8, 12, and 14 (Table 7), of which 174 showed homology at the protein level with Rhs1 and Rhs2 identified in *A. baumannii* AB307-0294 (Supplementary Table S6; Fitzsimons et al., 2018). We observed that while a group of 20 of these proteins belonging to the VGN14 showed an identity > 94% with the Rhs1 toxin of unknown function, 11 proteins (VGN12) were identical to the Rhs2 effector which carries an AHH nuclease domain (cl16862). This prompted us to analyze the variety of effector domains carried by the remaining Rhs-domain containing proteins (Supplementary Table S8). Remarkably, 59 candidates contained a Tox-GHH superfamily nuclease domain (cl21428) and 8 a YwqJ-deaminase superfamily domain (cl24268). Importantly, with the exception of the aforementioned PAAR-3 protein (ALH95481), none of these Rhs proteins bears a PAAR domain or a chaperone gene nearby, and thus their delivery mechanism remains unknown.

Diverse functions have been attributed to Rhs proteins in other bacteria such as motility, cellular toxicity, virulence in mice and insecticidal activity (Lien and Lai, 2017; Yang et al., 2018), and contact-dependent killing of other bacteria species in *A. baumannii* (Fitzsimons et al., 2018). Rhs proteins are capable of replacing the C-terminal end with a non-homologous alternative. By this means, they can switch between different toxin domains and thereby they have been included within the bacterial polymorphic toxin systems (Jamet and Nassif, 2015). Our findings indicate that the *rhs* genes associated with the *Acinetobacter vgrG* islands encode different C-terminal domains which might play different roles as toxins. To date none of these proteins have been implicated in *A. baumannii* virulence.

## Conclusion

The T6SS fulfills critical roles in bacterial competition, bacterium–host interaction and other functions associated with bacterial physiology. We therefore conducted a genomic comparative analyses of 191 *Acinetobacter* strains which let us identify two putative gene clusters responsible for the synthesis of a functional T6SS. The T6SS-1 was widespread in the genome of most species, while the T6SS-2 was restricted to three environmental species. In this respect, it will be interesting to determine whether the T6SS-2 is active and the roles it plays. Future work will certainly focus on the identification and characterization of the secreted components of this system.

The finding that the T6SS-1 gene cluster was present in both pathogenic and non-pathogenic species raises the hypothesis that the T6SS may have evolved to play different roles in the *Acinetobacter* lifestyle. In this respect, the variable regions associated with PAAR and *vgrG* genes could account for specialization of the T6SS based on the needs of each species. A plethora of toxin-encoding genes was detected in these genomic regions, with those encoding Rhs-domain containing proteins clearly overrepresented. Although no proteins showing significant homology to previously characterized effectors targeting eukaryotic cells (Lien and Lai, 2017) were detected, these regions also encode an impressive number of proteins of unknown function. Hence, we cannot rule out the existence of novel effector functions still to be discovered.

The overall observations described above not only highlight both the high diversity and potential plasticity of the *Acinetobacter* T6SS genes at the species level, but also support the notion that this system could have evolved differential and even strain-specific roles related to the interaction with other cells in particular environments. We hope the results obtained in this work can provide a foundation for future characterization of the *Acinetobacter* T6SSs and its effectors.

## Data Availability Statement

Publicly available datasets were analyzed in this study. This data can be found here: https://www.ncbi.nlm.nih.gov.

## Author Contributions

GR and SS conceived and designed the work. JS conducted the experimental work. GR and ME conducted the bioinformatic analysis. GR, ME, and SS analyzed the data and wrote the manuscript. All authors read and approved the final manuscript.

## Conflict of Interest

The authors declare that the research was conducted in the absence of any commercial or financial relationships that could be construed as a potential conflict of interest.

## Footnotes

1 http://www.endmemo.com/bio/gc.php

2 http://www.cbs.dtu.dk/services/TMHMM/

## Supplementary Material

FIGURE S1 Hcp secretion assay. The presence of Hcp (indicative of a functional T6SS) in concentrated culture supernatants of the indicated *Acinetobacter* strains grown up to stationary phase in L-Broth was determined by 18% SDS-PAGE and Coomasie Blue staining (see Repizo et al., 2015 for experimental details). MM, molecular markers.

FIGURE S2 Clustering of T6MC-proteins in 191 *Acinetobacter* strains. *A. baumannii* DSM30011 was used as reference. Strain subclustering according to T6MC classification is indicated. For *A. baumannii* strains, ST-classification according to the Pasteur scheme is also shown.

FIGURE S3 **(A–C)** Putative transmembrane domains present in *Acinetobacter* T6SS proteins with unknown function (Hyp1-3). TMHMM Server v.2.0 (http://www.cbs.dtu.dk/services/TMHMM) was used for predictions.

FIGURE S4 **(A)** TagN domain structure. The NCBI-CDD was used for the domain search (Marchler-Bauer et al., 2015). **(B)** Domain architecture of PAAR-3 proteins found in *Acinetobacter* spp. The NCBI-CDD database was used for the domain search (Marchler-Bauer et al., 2015).

FIGURE S5 Clustering of PAAR islands based on gene context. Conserved superfamily domains of proteins encoded within PAAR islands were used to define similar genetic contexts by clustering. Those gene islands sharing a similar genetic context were grouped in PAAR gene neighborhoods (PGN1-6, see Table 7 for detail). PAAR islands encoding putative toxins are indicated. Accession numbers corresponding to PAAR proteins encoded by *A. baylyi* ADP1 are boxed.

FIGURE S6 Clustering of VgrG islands based on gene context. Conserved superfamily domains of proteins encoded within VgrG islands were used to define similar genetic contexts by clustering. Those gene islands sharing a similar genetic context were grouped in VgrG gene neighborhoods (VGN1-14, see Table 7 for detail). PAAR islands encoding putative toxins are indicated. Accession numbers corresponding to VgrG proteins encoded by *A. baylyi* ADP1 are boxed.

TABLE S1 Strain under study.

TABLE S2 BlastP-homology search for VgrG domain-containing proteins in *Acinetobacter* spp.

TABLE S3 NCBI web-Conserved domains search tool results for *Acinetobacter* VgrG proteins.

TABLE S4 BlastP-homology search for PAAR proteins in the *Acinetobacter* genus.

TABLE S5 NCBI web-Conserved domains search tool results for *Acinetobacter* PAAR proteins.

TABLE S6 BlastP-homology search for toxins in PAAR and *vgrG* genetic islands in *Acinetobacter* spp.

TABLE S7 List of PAAR and *vgrG* proteins grouped into the gene neighborhoods defined in this study.

TABLE S8 NCBI web-Conserved domains search tool results for *Acinetobacter* Rhs proteins.
