# Supplementary figures and images for: Bioinformatic Analysis of the Type VI Secretion System and Its Potential Toxins in the Acinetobacter Genus
Source: Front Microbiol. 2019 Nov 1;10:2519. doi: 10.3389/fmicb.2019.02519 (PMC6838775; doi:10.3389/fmicb.2019.02519)

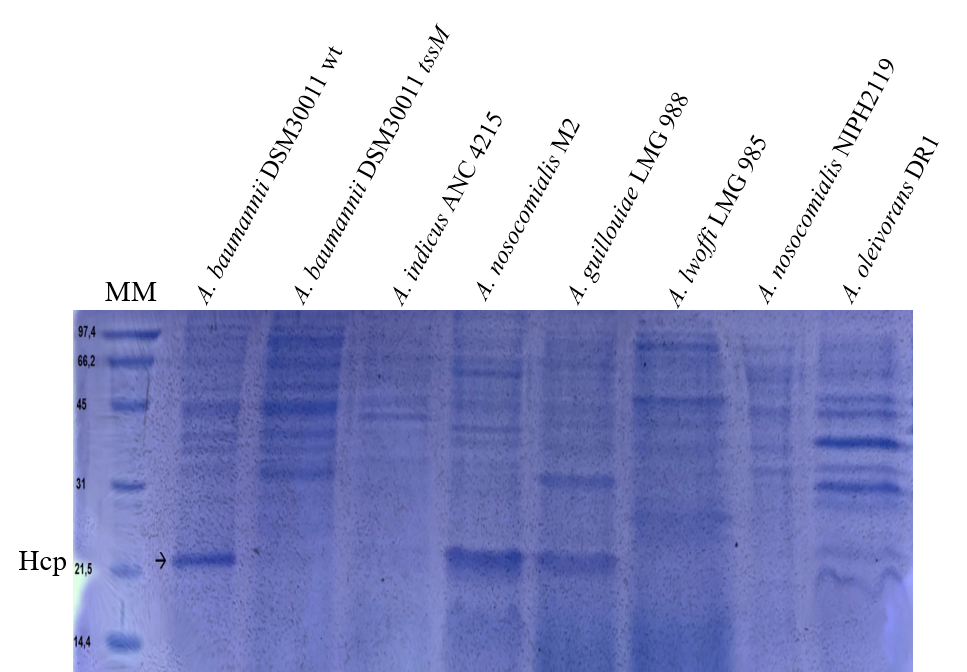

Supplement: FIGURE S1 — Hcp secretion assay. The presence of Hcp (indicative of a functional T6SS) in concentrated culture supernatants of the indicated Acinetobacter strains grown up to stationary phase in L-Broth was determined by 18% SDS-PAGE and Coomasie Blue staining (see Repizo et al., 2015 for experimental details). MM, molecular markers. [file Image_1.PNG]

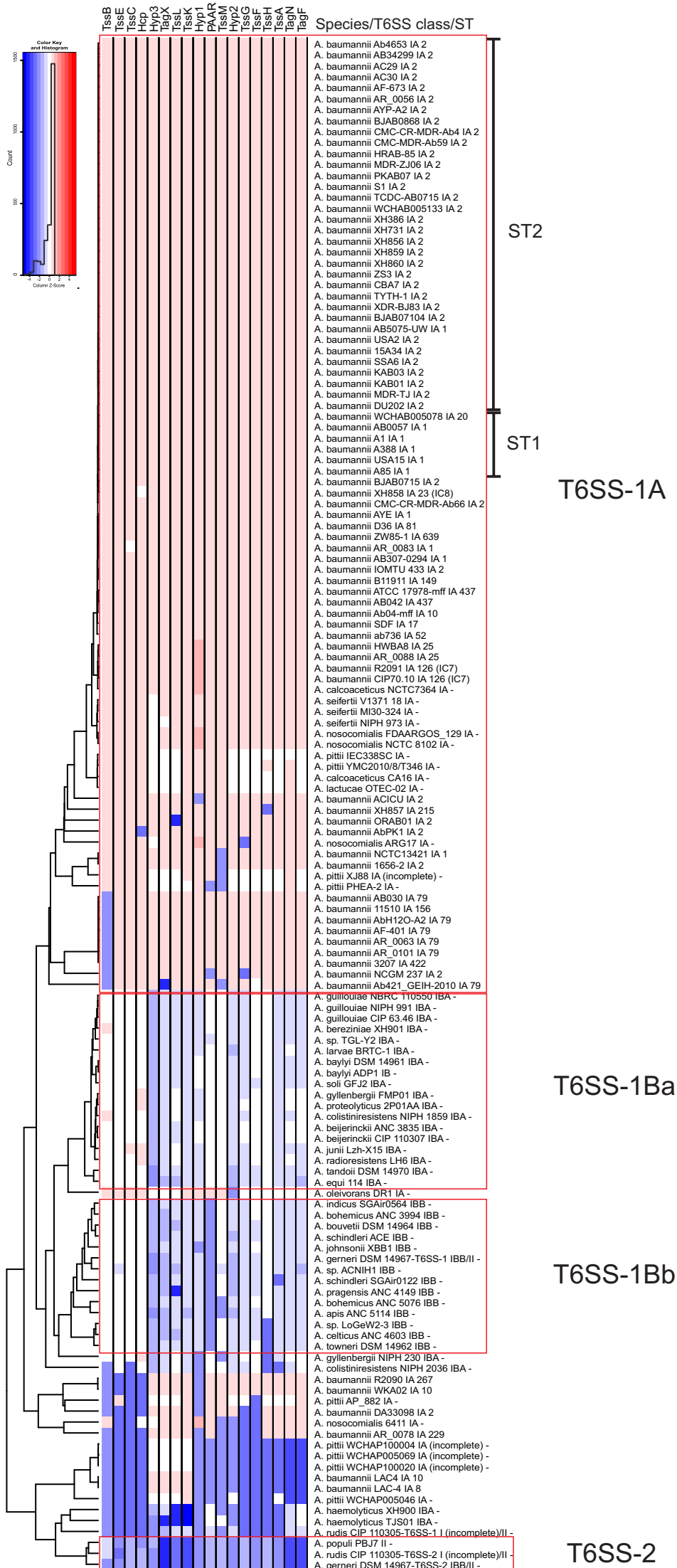

Supplement: FIGURE S2 — Clustering of T6MC-proteins in 191 Acinetobacter strains. A. baumannii DSM30011 was used as reference. Strain subclustering according to T6MC classification is indicated. For A. baumannii strains, ST-classification according to the Pasteur scheme is also shown. [file Image_2.PDF]

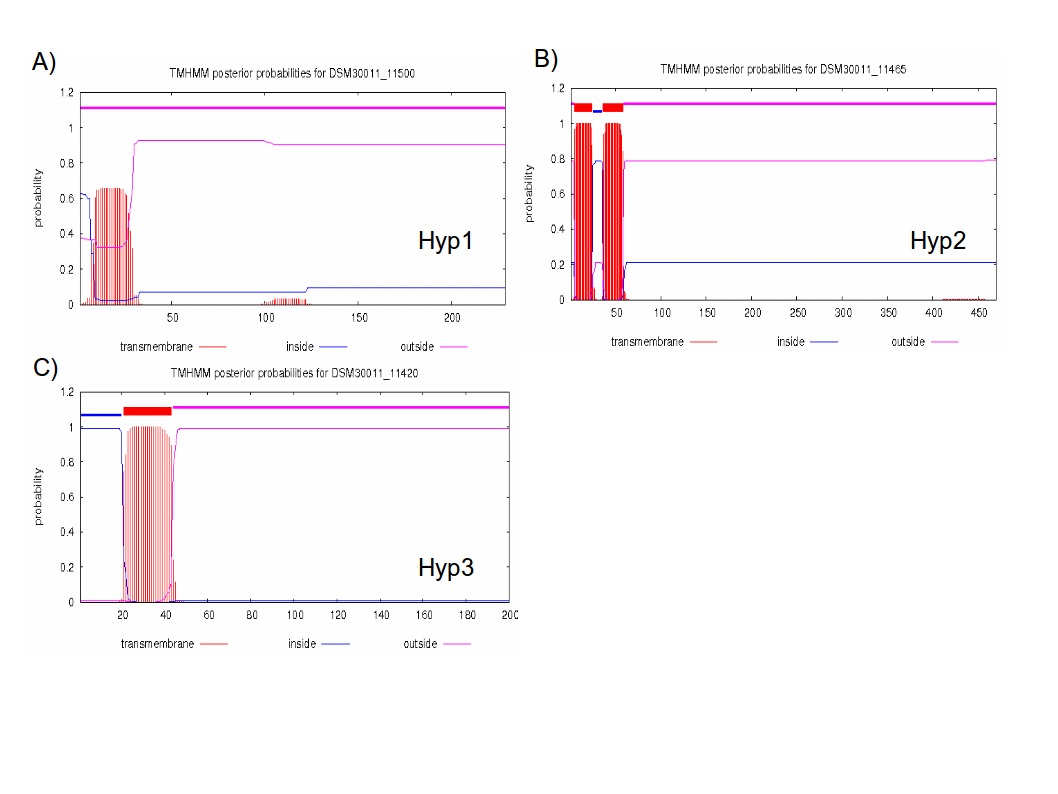

Supplement: FIGURE S3 — (A–C) Putative transmembrane domains present in Acinetobacter T6SS proteins with unknown function (Hyp1-3). TMHMM Server v.2.0 (http://www.cbs.dtu.dk/services/TMHMM) was used for predictions. [file Image_3.TIF]

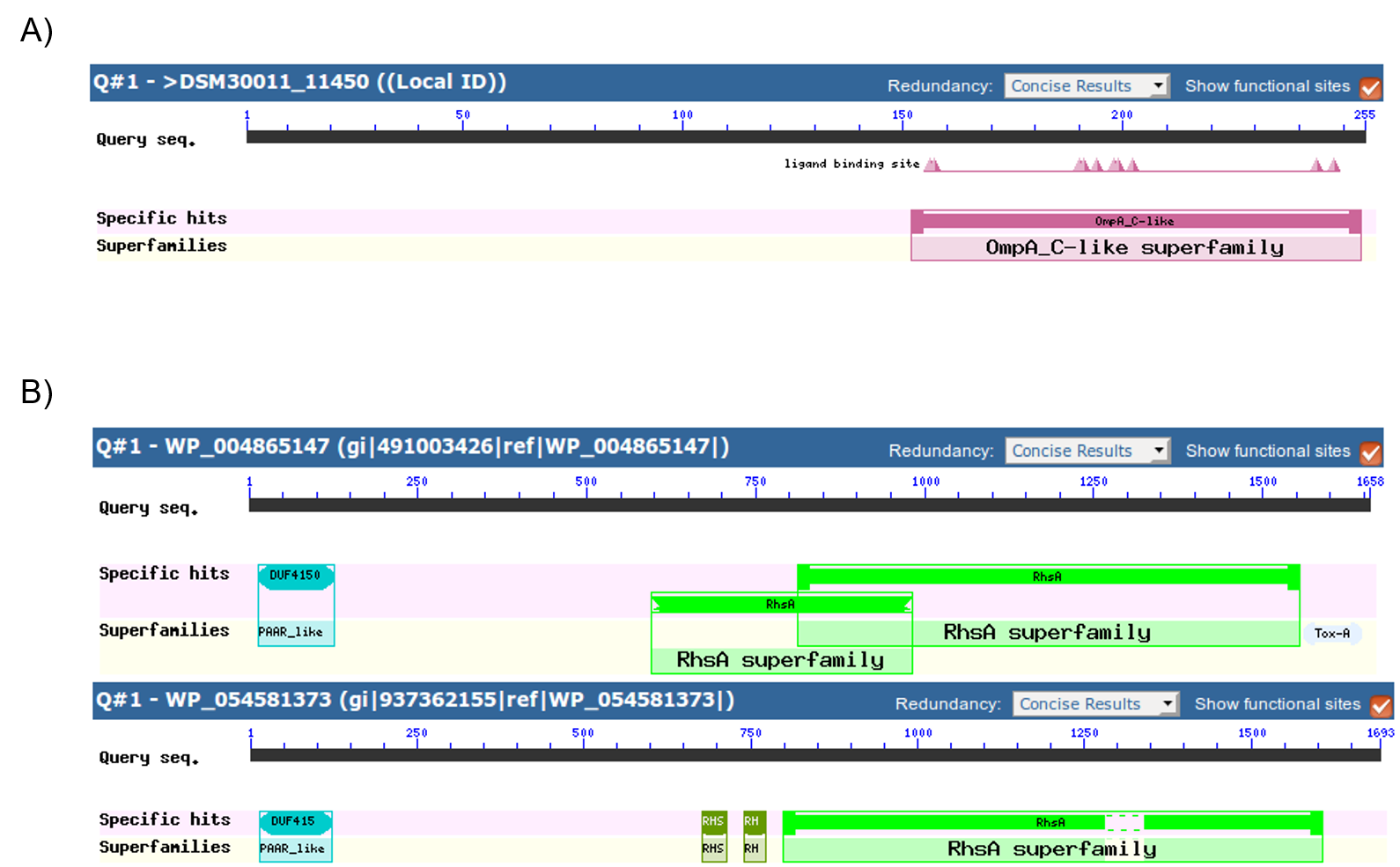

Supplement: FIGURE S4 — (A) TagN domain structure. The NCBI-CDD was used for the domain search (Marchler-Bauer et al., 2015). (B) Domain architecture of PAAR-3 proteins found in Acinetobacter spp. The NCBI-CDD database was used for the domain search (Marchler-Bauer et al., 2015). [file Image_4.TIF]
