# Supplementary material for: Bioinformatic Analysis of the Type VI Secretion System and Its Potential Toxins in the Acinetobacter Genus
Source: Front Microbiol. 2019 Nov 1;10:2519. doi: 10.3389/fmicb.2019.02519 (PMC6838775; doi:10.3389/fmicb.2019.02519)
Supplement: FIGURE S5 — Clustering of PAAR islands based on gene context. Conserved superfamily domains of proteins encoded within PAAR islands were used to define similar genetic contexts by clustering. Those gene islands sharing a similar genetic context were grouped in PAAR gene neighborhoods (PGN1-6, see Table 7 for detail). PAAR islands encoding putative toxins are indicated. Accession numbers corresponding to PAAR proteins encoded by A. baylyi ADP1 are boxed. [file Image_5.PDF]

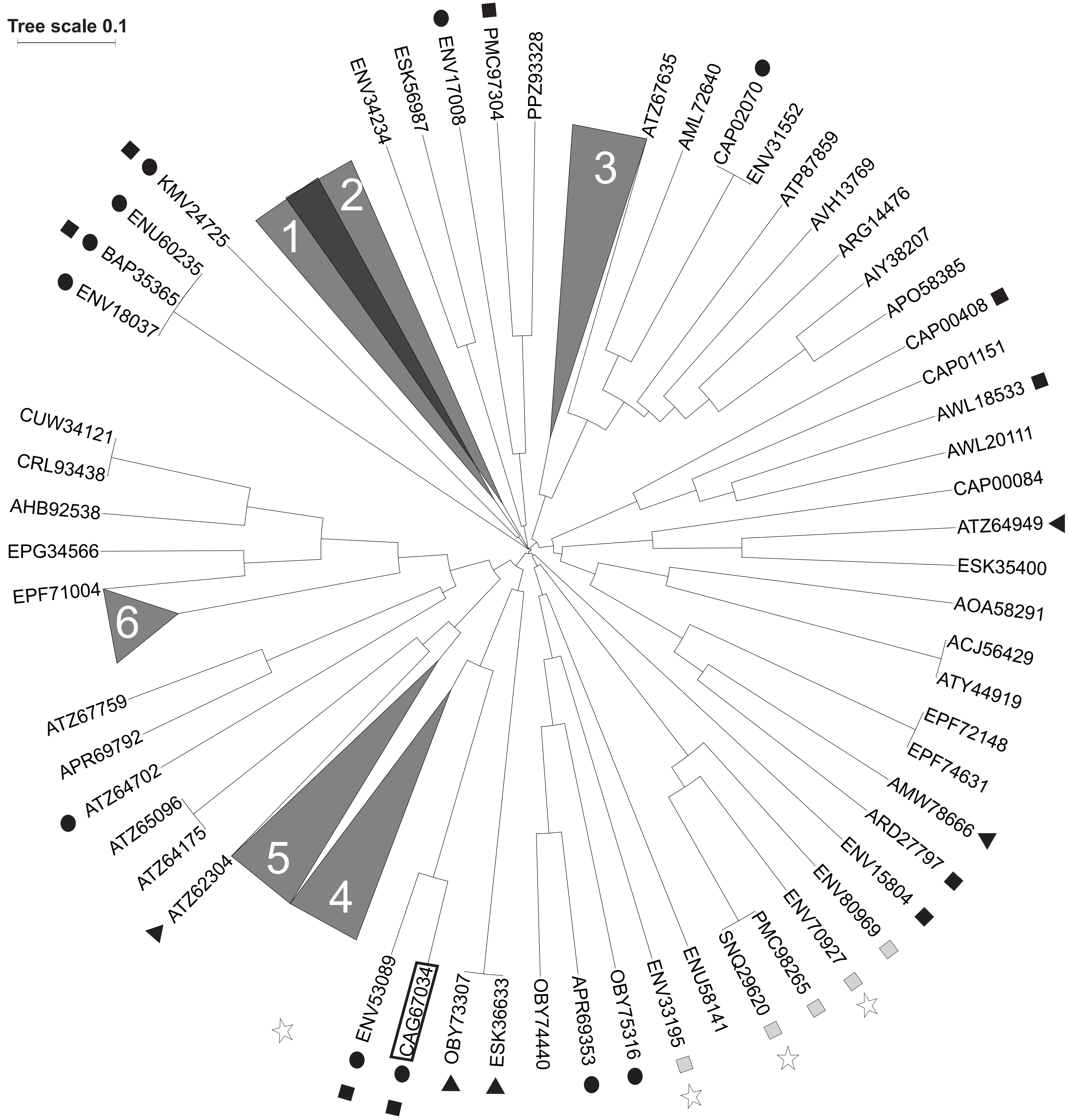

● PAAR-5 and PAAR-2 genes in tandem

☆ PAAR gene encoded by a T6SS-1Bb[+] *Acinetobacter* spp.

▲ PAAR gene next to a VRR-domain nuclease gene

■ (grey) PAAR gene in a *vgrG* island

■ PAAR gene next to a Tpe1-toxin gene
