# Supplementary material for: Bioinformatic Analysis of the Type VI Secretion System and Its Potential Toxins in the Acinetobacter Genus
Source: Front Microbiol. 2019 Nov 1;10:2519. doi: 10.3389/fmicb.2019.02519 (PMC6838775; doi:10.3389/fmicb.2019.02519)
Supplement: FIGURE S6 — Clustering of VgrG islands based on gene context. Conserved superfamily domains of proteins encoded within VgrG islands were used to define similar genetic contexts by clustering. Those gene islands sharing a similar genetic context were grouped in VgrG gene neighborhoods (VGN1-14, see Table 7 for detail). PAAR islands encoding putative toxins are indicated. Accession numbers corresponding to VgrG proteins encoded by A. baylyi ADP1 are boxed. [file Image_6.PDF]

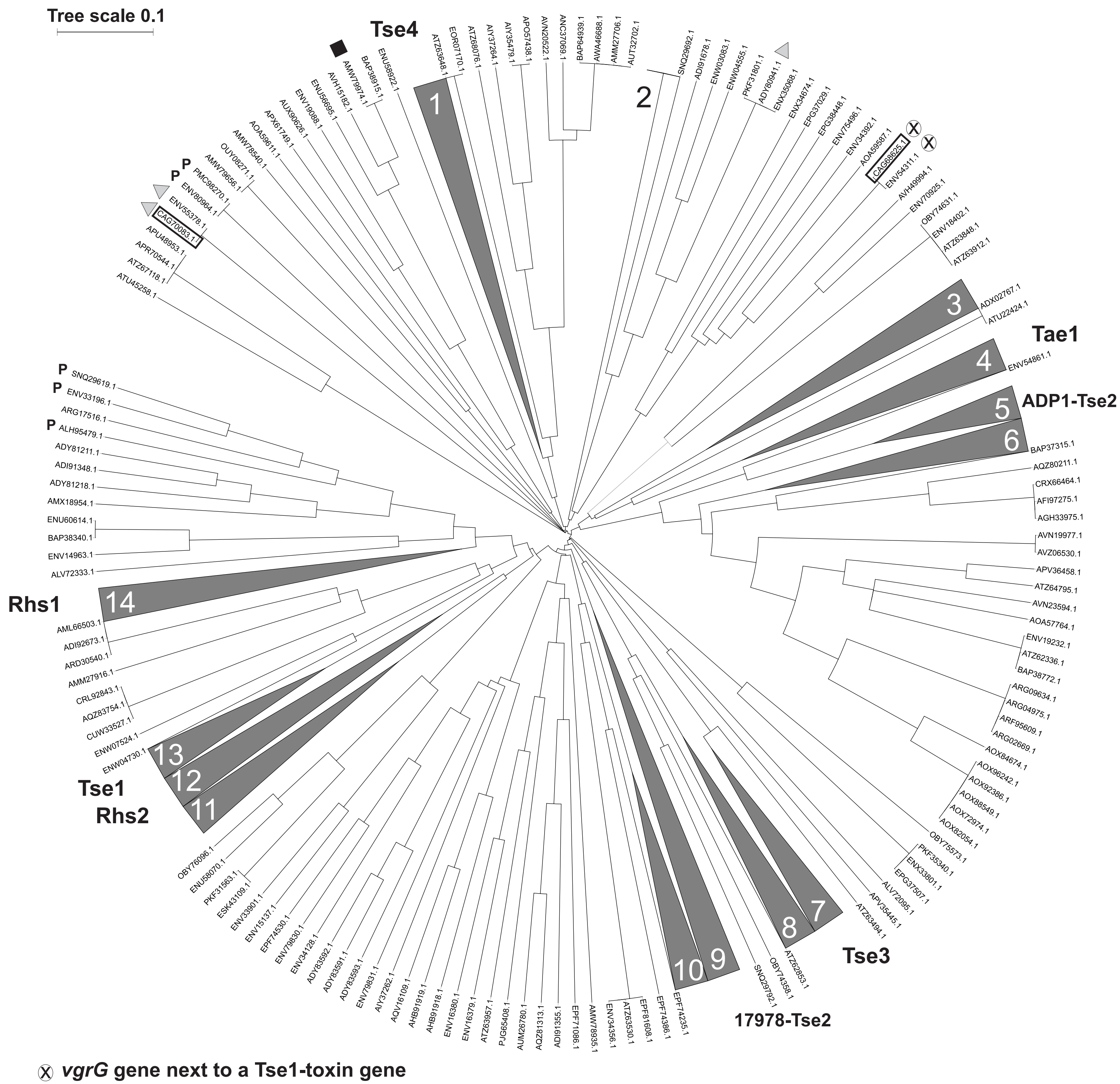

Ⓧ *vgrG* gene next to a Tse1-toxin gene

▲ *vgrG* gene next to a Tle1-toxin gene

P *vgrG*-PAAR island

■ *vgrG* gene next to a Tpe1-toxin gene
